# Decorin Is a Newly Discovered Target of *Akkermansia muciniphila* in the Treatment of Sepsis‐Associated Encephalopathy

**DOI:** 10.1111/cns.70642

**Published:** 2025-11-04

**Authors:** Bingqing Gong, Feixiang Li, Yuanyuan Bai, Yang Guo, Rui Zhang, Yonghao Yu, Beibei Dong

**Affiliations:** ^1^ Department of Anesthesiology Tianjin Medical University General Hospital Tianjin China; ^2^ Tianjin Institute of Anesthesiology Tianjin China; ^3^ Department of Anesthesiology, Beijing Chao‐Yang Hospital Capital Medical University Beijing China

**Keywords:** *Akkermansia muciniphila*, autophagy, decorin, sepsis, sepsis‐associated encephalopathy

## Abstract

**Background:**

Sepsis‐associated encephalopathy (SAE) is a major contributor to mortality in septic patients, with its mechanisms being incompletely understood and effective therapies lacking. While the gut bacterium *Akkermansia muciniphila* (AKK) has shown beneficial effects in systemic infections, its specific role and mechanisms in SAE remain undefined.

**Methods:**

We investigated the role of AKK in a murine model of sepsis induced by cecal ligation and puncture (CLP). Oral administration of AKK was confirmed via 16S rRNA sequencing. Mice underwent behavioral assessments to evaluate cognitive function. Hippocampal levels of pro‐inflammatory cytokines were measured, and transcriptomic profiling was performed to identify key mediators. Functional analyses were conducted to delineate the role of the identified protein, Decorin (DCN), in neuronal survival, apoptosis, and autophagy. Simultaneously, we intervened in the expression of mouse DCN using AAV virus and further validated.

**Results:**

Oral AKK pretreatment was successfully enriched in the gut and significantly ameliorated CLP‐induced cognitive deficits. It concurrently reduced hippocampal pro‐inflammatory cytokine levels through a robust, DCN‐independent anti‐inflammatory pathway. Transcriptomics revealed that AKK treatment notably upregulated hippocampal DCN expression. Functional studies demonstrated that DCN contributed to AKK's neuroprotective effects by promoting autophagy and suppressing neuronal apoptosis, representing a distinct mechanism of action.

**Conclusion:**

Our findings reveal a dual mechanism through which AKK mitigates SAE: (1) suppression of hippocampal inflammation via a DCN‐independent pathway, and (2) DCN‐dependent modulation of neuronal apoptosis and autophagy. This study establishes AKK as a promising microbial intervention for SAE and identifies DCN as a context‐specific mediator of its neuroprotective effects.

## Graphical abstract

*Akkermansia muciniphila* protects against sepsis‐associated encephalopathy by reducing hippocampal inflammation and modulating neuronal survival through decorin‐dependent and ‐independent pathways, highlighting its potential as a microbial therapy.

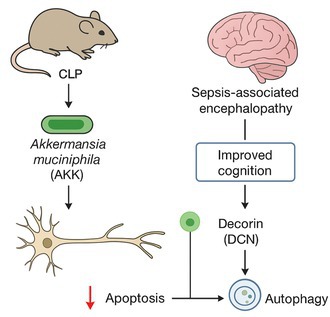

## 1 Introduction

Sepsis is defined as life‐threatening organ dysfunction caused by dysregulation of systemic inflammatory and immune responses during host infection [1]. Sepsis‐associated encephalopathy (SAE) is defined as a diffuse cerebral dysfunction occurring during sepsis, in the absence of direct central nervous system infection, structural brain lesions, or other identifiable encephalopathies. Its pathophysiological mechanisms are thought to involve neuroinflammation, ischemia–hypoxia, and disruption of the blood–brain barrier [2, 3]. Clinically, SAE is frequently characterized by acute cognitive dysfunction and often manifests early in the course of sepsis [4], affecting up to 70% of patients. It is strongly associated with increased mortality in intensive care units, while survivors commonly experience persistent cognitive deficits, including memory impairment, attention difficulties, and reduced speech fluency [5]; as well as psychological disorders such as depression, anxiety, and post‐traumatic stress disorder [6]. The most prominent manifestations of SAE are cognitive deficits, particularly impairments in learning, memory, attention, and delirium [2]. The hippocampus, a central hub for episodic memory, spatial navigation, and learning [7], is particularly vulnerable, and neuronal loss in this region may be a key contributor to SAE‐related cognitive decline [8] At present, no clear diagnostic criteria or specific treatment strategies for SAE exist, underscoring the urgent need for novel therapeutic approaches. The gut–brain axis, a bidirectional communication network linking the gastrointestinal tract and central nervous system through neural, endocrine, and immune pathways [9], has attracted increasing attention. Recent evidence indicates that gut microbiota dysbiosis may contribute to the development of SAE via the gut–brain axis [10].

*Akkermansia muciniphila* (AKK) is an anaerobic, Gram‐negative bacterium with demonstrated probiotic potential in a variety of diseases, including immune and metabolic disorders [11, 12]. It has been shown to ameliorate metabolic dysfunctions such as obesity and diabetes in both humans and mice [13, 14]. More recently, AKK was reported to protect against sepsis and sepsis‐induced injury in multiple organs, including the lungs, kidneys, and liver [15, 16]. However, its role in the development and pathophysiological mechanisms of SAE remains unclear.

Autophagy and apoptosis are two fundamental processes determining cell fate under stress [17]. Their dynamic interplay—shaped by the type, intensity, and duration of stress—critically influences whether neurons survive or undergo programmed death [18, 19]. Dysregulation of the autophagy–apoptosis balance has been implicated in several neurological diseases, yet direct evidence for such alterations in the hippocampus during SAE remains limited. Notably, pyramidal neurons in the CA1 region of the hippocampus exhibit poor tolerance to ischemia, hypoxia, oxidative stress, and inflammation, making them among the most vulnerable neuronal populations in the brain [20, 21]. Given the strong association between hippocampal neuronal integrity and cognitive function, understanding how autophagy and apoptosis are regulated in SAE may provide crucial insights into disease mechanisms and therapeutic targets.

In this study, we investigated the role of AKK in modulating hippocampal injury in septic mice, with a particular focus on DCN‐mediated regulation of autophagy and apoptosis. Our results demonstrate that AKK supplementation alleviates sepsis‐induced cognitive dysfunction, reduces hippocampal neuroinflammation, enhances autophagy, inhibits apoptosis, and ultimately mitigates SAE progression, suggesting that AKK may represent a promising novel therapeutic strategy.

## 2 Materials and Methods

### 2.1 Murine Model

Male specific pathogen‐free C57BL/6J mice (6–8 weeks, Laboratory Animal Center, Military Medical Science Academy, Beijing, China) were maintained under controlled conditions (20°C–25°C, 55%–65% humidity, 12‐h light/dark cycle) with free access to food and water. Mice were acclimatized for ≥ 10 days before surgery. All procedures were approved by the Animal Experiment Ethics Committee of Tianjin Medical University General Hospital (IRB2025‐DW‐15).

### 2.2 Caecal Ligation and Puncture (CLP)‐Induced Sepsis

Cecal ligation and puncture (CLP) is the gold standard for inducing sepsis in mice and reliably produces both short‐ and long‐term behavioral deficits [15]. Mice were anesthetized with 3% sevoflurane, and a ~1 cm incision was made in the lower left abdomen to expose the cecum. The cecum was ligated with 6–0 silk suture at the midpoint between the ileocecal valve and the cecal tip, punctured once with a 21G needle, and a small amount of fecal content was extruded. The cecum was then returned to the abdominal cavity to induce moderate‐to‐severe sepsis. Sham‐operated controls underwent laparotomy without ligation or puncture. All mice received subcutaneous saline (50 mL/kg) immediately after surgery to compensate for fluid loss. Postoperative behavior and survival were monitored.

### 2.3 Preprocessing

*Akkermansia muciniphila* (AKK, BNCC341917) was obtained from BNCC (China) and anaerobically cultured in liquid thioethanol saline medium (BNCC353538). The bacterial suspension was adjusted to 2 × 10^9^ CFU/mL. Mice were randomly assigned to four groups: sham, sham + AKK, CLP, and CLP + AKK. Beginning 3 days before surgery (Day −3), mice received daily intragastric administration of 300 μL AKK suspension or an equal volume of sterile saline using a No. 12 gavage needle, continued for 7 days after surgery. Tissue samples were collected immediately after surgery, and behavioral tests were performed on Days 3 and 7 post‐surgery.

### 2.4 16S rRNA Gene Sequencing

Fecal samples were collected 24 h after surgery from each group. Microbial DNA was extracted, and the V3–V4 hypervariable regions of the bacterial 16S rRNA gene were amplified by PCR. Amplicons were sequenced on an Illumina MiSeq platform (PE300), and data were processed by Majorbio Bio. Optimized sequences were clustered into operational taxonomic units (OTUs) at 97% similarity using UPARSE 11, with the most abundant sequence in each OTU selected as the representative. Taxonomic classification was assigned using the RDP classifier with a confidence threshold of 0.7 against the Silva v138 database. Alpha diversity indices (Chao, Shannon, and Simpson) were calculated in Mothur v1.30.2. Principal coordinate analysis (PCoA) based on Bray–Curtis dissimilarity was performed using the Vegan v2.4.3 package.

### 2.5 Transcriptomics

#### 2.5.1 RNA Extraction and Library Preparation

Total RNA was extracted from frozen hippocampal tissue using the RNeasy Mini Kit in combination with TRIzol reagent. RNA purity and concentration were measured with a spectrophotometer (Thermo Scientific, USA), and integrity was assessed using an Agilent 2100 Bioanalyzer (RIN > 6.0; 28S/18S ≥ 0.7). Libraries were prepared with the NEBNext Ultra RNA Library Prep Kit following the manufacturer's protocol.

#### 2.5.2 RNA Sequencing and Differential Expression Analysis

Libraries were sequenced on the Illumina NovaSeq 5000 platform to generate 150‐bp paired‐end reads, yielding 45.18–49.38 Mb raw reads per sample. Low‐quality reads were filtered with Trimmomatic to obtain 44.21–48.31 Mb clean reads. Reads were aligned to the mouse genome (GRCm38.p6) using HISAT2. Gene expression levels were quantified as fragments per kilobase of transcript per million mapped reads (FPKM), and read counts were obtained with HTSeq. Differentially expressed genes (DEGs) were identified using the DESeq R package (2012) with thresholds of *p* < 0.05 and fold change > 1.0. Hierarchical clustering was performed to visualize expression patterns among groups. Gene Ontology (GO) and Kyoto Encyclopedia of Genes and Genomes (KEGG) enrichment analyses were conducted based on hypergeometric distribution using R and OECloud tools (https://cloud.oebiotech.cn). Gene set enrichment analysis (GSEA; http://software.broadinstitute.org/gsea/index.jsp) was also performed to identify signaling pathways associated with SAE.

#### 2.5.3 Reverse Transcription and Quantitative PCR

RNA isolation and RT–qPCR were conducted as described above. Briefly, total RNA was extracted with TRIzol, and 1 μg RNA was reverse‐transcribed into cDNA using a commercial reverse transcription kit. Quantitative PCR was performed using 2× Power SYBR Green with LightCycler conditions. Relative gene expression was normalized to GAPDH or β‐actin and calculated using the 2^−Δ*C*t^ method. Primer sequences are available upon request.

### 2.6 Behavioral Tests

#### 2.6.1 Open Field Test (OFT)

The OFT was conducted to assess activity, exploratory behavior, and memory. The apparatus was a 60 × 60 × 50 cm enclosed area, in which the floor was divided into nine equally sized squares by black lines. During the training phase, the mice were placed in the lower left quadrant, and allowed to explore the area for 5 min. During the testing phase, the numbers of line crossings and entries into the central region were automatically recorded for each mouse. High numbers of line crossings and entries into the central region indicated good motor performance and low levels of anxiety.

#### 2.6.2 Morris Water Maze (MWM) Test

On the 7th day after surgery, the MWM test was performed to evaluate the spatial learning and memory of the mice. In accordance with previously reported methods, we used a computer video tracking system to record the movements of the mice in a water maze. In brief, a transparent circular platform was placed in a fixed quadrant of a circular pool 1 cm below the water surface. During the training period, the mice were first placed on the platform for 30 s to adapt to the environment and then released into the water. In each experiment, the mice were allowed to search for the platform for a maximum of 60 s. If a mouse failed to find the platform within 60 s, it was guided to the platform and allowed to stay on it for 30 s. All the mice were subjected to four training trials per day for 5 days, and the latency to find the platform was recorded in each trial. One hour after the last training trial on the 5th day of training, the platform was removed from the water maze. The mice were then placed in the water from the quadrant in which the platform had been located facing the pool wall. The movement trajectories of the mice over 60 s were recorded. The memory of the mice was evaluated by determining the number of platform crossings.

### 2.7 Histopathological Staining

The mice were anesthetized, and the hearts were perfused with 4% paraformaldehyde and phosphate‐buffered saline (PBS). Brain tissue was removed, fixed in 4% paraformaldehyde for 72 h, and then embedded in paraffin. Brain tissue slices (5 μm) were subjected to Nissl staining and hematoxylin and eosin (HE) staining.

### 2.8 Immunofluorescence Staining

The expression of DCN, P62, and LC3 in hippocampal neurons was measured via immunofluorescence. Frozen sections were prepared and blocked with 3% bovine serum albumin containing Triton X‐100 for 2 h. The samples were incubated with primary antibody (anti‐DCN antibody, 1:200 dilution; anti‐LC3 antibody, 1:200 dilution; anti‐P62 antibody, 1:500 dilution) at 4°C overnight and then with secondary antibody (CY3‐conjugated goat anti‐rat IgG, 1:400 dilution; FITC‐conjugated goat anti‐rabbit IgG, 1:400 dilution) at 37°C for 1 h. The cell nuclei were labeled with a sealing agent containing DAPI. For terminal deoxynucleotide transferase dUTP nick end labeling (TUNEL) staining, frozen sections were fixed with 4% paraformaldehyde for 15 min and washed three times with PBS. Finally, the slices were incubated with 0.1% Triton X‐100 for 5 min and stained with an in situ cell death detection kit (Beyotime, China) according to the manufacturer's instructions.

The sections were subsequently independently examined using an inverted fluorescence microscope.

### 2.9 Western Blotting

Twenty‐four hours after surgery, hippocampal tissues from mice in each group were collected and weighed, and protein was extracted using commercial lysis buffer (#P0013B, Beyotime) containing protease inhibitors (#P1048, Beyotime, China) and phosphatase inhibitors (#P1082, Beyotime, China). The samples were added to loading buffer (Beijing Solarbio Science & Technology Co. Ltd.), boiled and denatured. The protein samples were subsequently separated on 10% or 12% SDS‐polyacrylamide gels and transferred to a polyvinylidene fluoride (PVDF) membrane. The PVDF membrane was blocked with 5% skim milk at room temperature for 2 h. After blocking, the membrane was incubated overnight on a shaker at 4°C with primary antibodies against the following proteins: ATG5, P62, decorin, and LC3 (1:1000 dilution; Cat Nos: 10181‐2‐AP, 18420‐1‐AP, 66847‐1‐lg, and 14600‐1‐AP; Proteintech, China), BAX and BCL‐2 (Abcam, ab32503, ab182858, Britain). The next day, the PVDF membrane was washed three times with TBST, incubated at room temperature with goat anti‐rabbit (1:5000 dilution; Cat No 31,466, Invitrogen, USA) or anti‐mouse (1:5000 dilution; Cat No. 31,430, Invitrogen, United States) antibodies and washed in TBST for 1 h. Finally, the protein bands were visualized with enhanced chemiluminescence (ECL) reagents (Cat No P0018AM, Beyotime Biotechnology, China), and the data were analyzed using ImageJ 20.

### 2.10 Transmission Electron Microscopy

Hippocampal tissue was collected 24 h after surgery and fixed in 2.5% glutaraldehyde solution at 4°C for 24 h. The tissue was subsequently incubated with acetone in water and embedded in Epon. Ultrathin sections were cut, and the cell structure was observed via transmission electron microscopy.

### 2.11 Intracranial Injection of Adenovirus

Recombinant AAV9 vectors carrying mouse Dcn shRNA (AAV‐DCN) or a control sequence (AAV‐Control) were purchased from Obio Technology (Shanghai, China). The shRNA sequence targeting Dcn mRNA was CCTGTCTAAGAACCAACTAAAGAAGTCGTGAGAAGTAGAA.

Mice received stereotaxic microinjections of AAV‐DCN or AAV‐Control into the hippocampal lateral ventricles using a Hamilton microinjector (10 μL) at the following coordinates relative to bregma: AP = −1.1 mm, ML = +1.5 mm, DV = −3.5 mm. A total of 1.04 × 10^10^ genome copies were delivered bilaterally at 0.2 μL/min (0.3 μL per side). After injection, the syringe was left in place for 10–15 min to ensure diffusion and prevent backflow. On Day 21 post‐injection, a cecal ligation and puncture (CLP) model was established for subsequent behavioral and histological analyses [16].

### 2.12 Statistical Analysis

Unless otherwise specified, the data in this study are presented as the means ± standard deviations. The experimental data were analyzed via two‐way unpaired Student's *t* tests or one‐way analysis of variance (ANOVA). Two‐way ANOVA was used for statistical analysis of the MWM test data. Statistical analysis of the relative abundance of AKK in mouse feces was conducted using ANCOM. The Wilcoxon rank sum test was used to analyze the abundance of microbes at the family and genus levels in the feces. The specific statistical methods used are provided in the figure legends. All the data in this study were analyzed using GraphPad Prism V.10. *p* < 0.05 was considered to indicate statistical significance, whereas *p* > 0.05 was considered to indicate no statistical significance.

## 3 Results

### 3.1 AKK Attenuates Cognitive Decline and Reduce Inflammationin SAE

**FIGURE 1:**
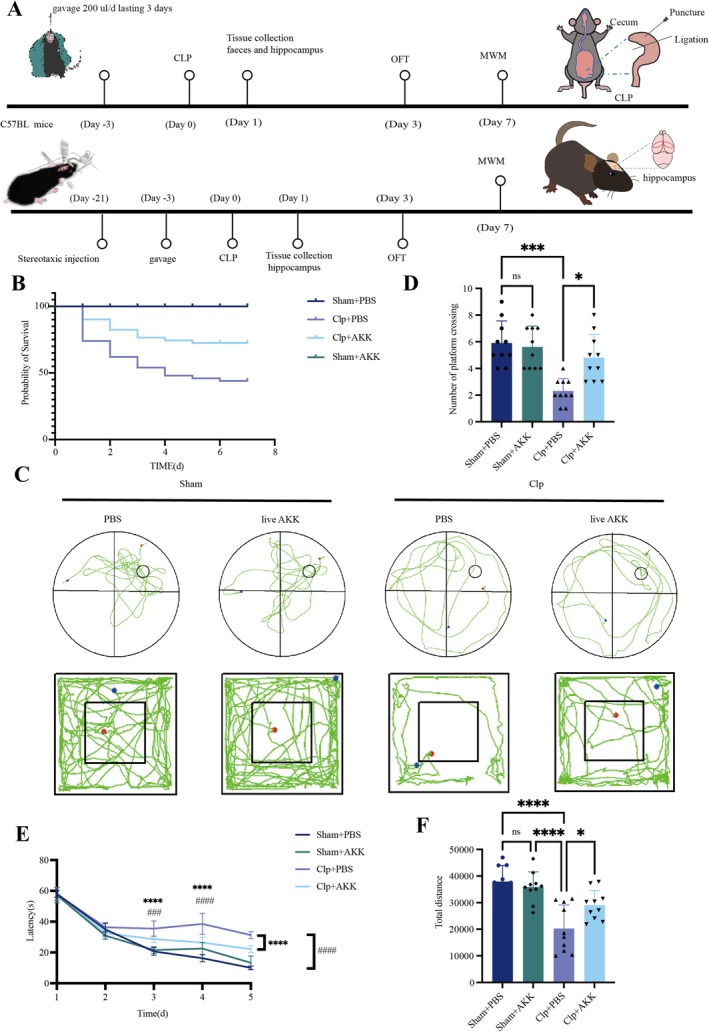
Early AKK treatment ameliorates cognitive impairment caused by sepsis. (A) Schematic illustration of the experimental design. (B) Mice were orally administered PBS or live AKK (200 μL each time) for 3 days (once daily, surgery was performed 2 h after the third treatment), followed by CLP or sham surgery. Kaplan–Meier curves were used to determine the survival rate (*n* = 50 mice per group). (C) Trajectory diagrams of the mice in the MWM test and OFT (*n* = 10). (D) Number of platform crossings during postoperative probe phase of the MWM test. (E) Escape latency of the mice during the training phase of the MWM test. (F) Total distance traveled by each group of mice in the OFT. The data are presented as the means ± SD. *p < 0.05, **p < 0.01, ***p < 0.001, and ****p < 0.0001 (*n* = 10 mice per group).

**FIGURE 2:**
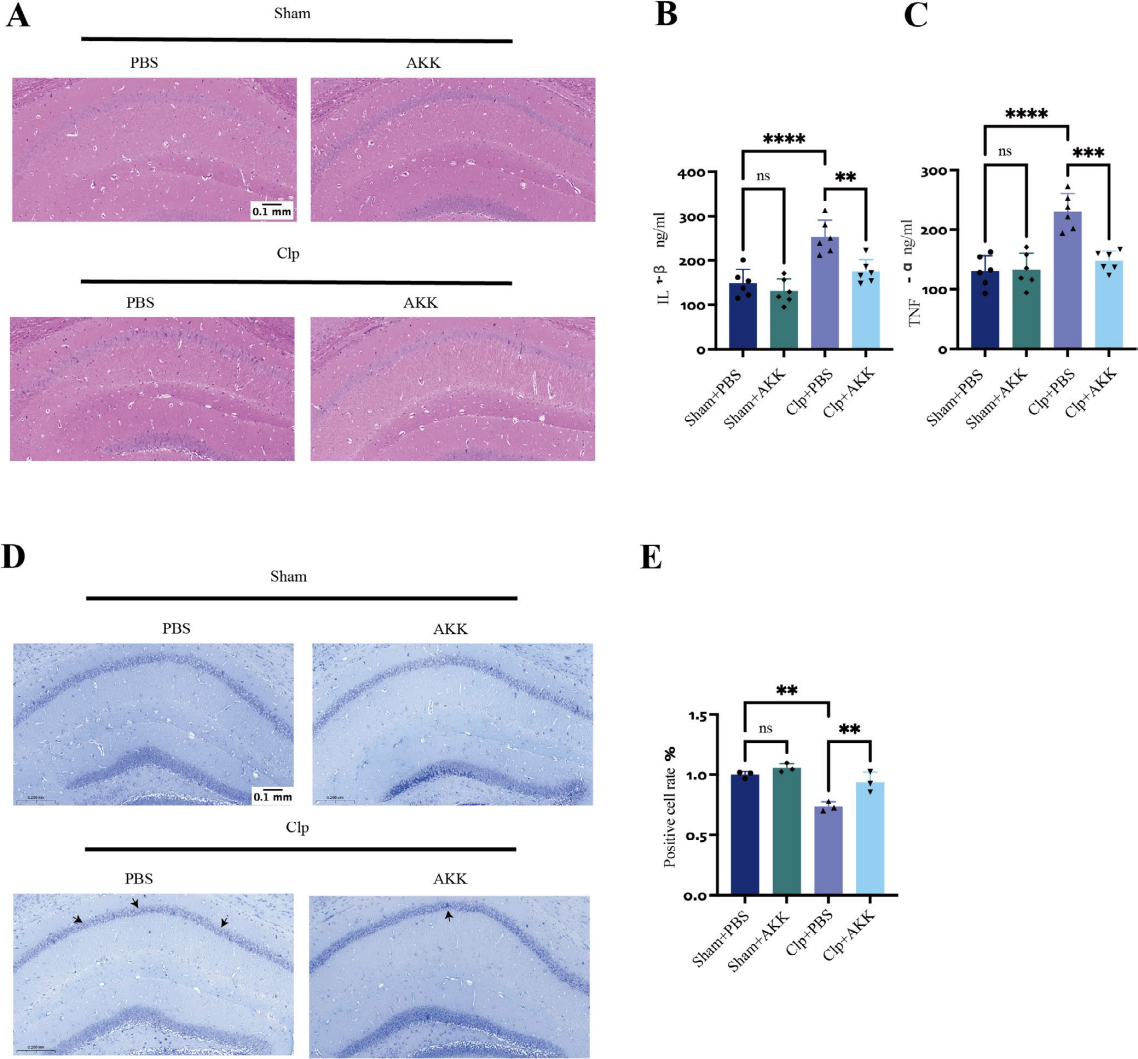
AKK may reduce cell damage in the hippocampal CA1 region in CLP model mice. (A) Representative H&HE‐stained images (scale: 0.1 mm, magnification: ×20) (*n* = 3). (B, C) Measurement of IL‐1β and TNF‐α levels in the hippocampus of sham group and CLP model mice treated with PBS and AKK (*n* = 6). (D) Nissl‐stained images of the hippocampal CA1 region of mice. (scale: 0.1 mm, magnification: ×20) (*n* = 3). (E) Quantitative analysis of Nissl staining. The data are presented as the means ± SD. *p < 0.05, **p < 0.01, ***p < 0.001, and ****p < 0.0001.

CLP was used to establish the SAE model, as 24 h post‐surgery represents a critical period for systemic inflammation and sepsis‐induced brain pathology [17]. Accordingly, all samples were collected at this time point. Continuous supplementation with *Akkermansia muciniphila* (AKK) for 3 days prior to surgery has been reported to exert protective effects [15]; therefore, the same regimen was adopted. Mice pretreated with PBS or AKK were subjected to sham surgery or CLP (Figure 1A). The survival rate was markedly reduced in the CLP group compared with sham controls, confirming successful model induction. AKK pretreatment modestly prolonged survival relative to PBS‐pretreated CLP mice (Figure 1B). On Day 3 post‐surgery, open‐field testing revealed reduced locomotor activity in CLP + PBS mice compared with sham controls, whereas AKK pretreatment significantly increased total distance traveled (*F*(3,36) = 8.337, *p* < 0.0001; *F*(3,36) = 4.117, *p* = 0.0299) (Figure 1C,F). On Day 7, Morris water maze testing further showed prolonged escape latencies in CLP mice compared with sham, which were significantly shortened in AKK‐pretreated CLP mice (Day 3, *F*(4,180) = 13.05, *p* < 0.0001; Day 4, *F*(3,36) = 19.71, *p* < 0.0001; Day 5, *F*(3,36) = 18.79, *p* < 0.0001; Day 3, *F*(3,36) = 5.969, *p* = 0.0002; Day 4, *F*(3,36) = 10.70, *p* < 0.0001; Day 5, *F*(3,36) = 8.060, *p* < 0.0001). In the probe trial, CLP mice exhibited significantly fewer platform crossings, while AKK pretreatment increased crossing numbers compared with PBS (*F*(3,36) = 4.100, *p* = 0.0002; *F*(3,36) = 2.834, *p* = 0.0276) (Figure 1C–E). These results demonstrate that sepsis reduces locomotor activity and impairs learning and memory, whereas AKK ameliorates these deficits. Histological and biochemical analyses corroborated the behavioral findings. In the hippocampal CA1 region, CLP caused neuronal damage characterized by altered morphology, disorganized arrangement, and darkly stained neurons (Figure S3A). Levels of IL‐1β and TNF‐α were also significantly increased (IL‐1β, *F*(3,20) = 4.550, *p* = 0.0207; TNF‐α, *F*(3,20) = 9.466, *p* < 0.0001) (Figure 2B,C). AKK pretreatment reduced neuronal abnormalities, preserved cellular organization, and lowered IL‐1β and TNF‐α expression (IL‐1β, *F*(3,20) = 7.481, *p* = 0.0002; TNF‐α, *F*(3,20) = 7.846, *p* = 0.0001). Consistently, Nissl staining showed that CLP markedly increased neuronal loss, which was significantly attenuated by AKK (*F*(3,8) = 8.753, *p* = 0.0012; *F*(3,8) = 6.673, *p* = 0.0066) (Figure 2D,E).

Together, these findings indicate that CLP induces hippocampal inflammation, neuronal injury, and behavioral deficits characteristic of SAE, while early AKK supplementation effectively mitigates these pathological changes.

### 3.2 Involvement of AKK in the Intestines in SAE in Mice

**FIGURE 3:**
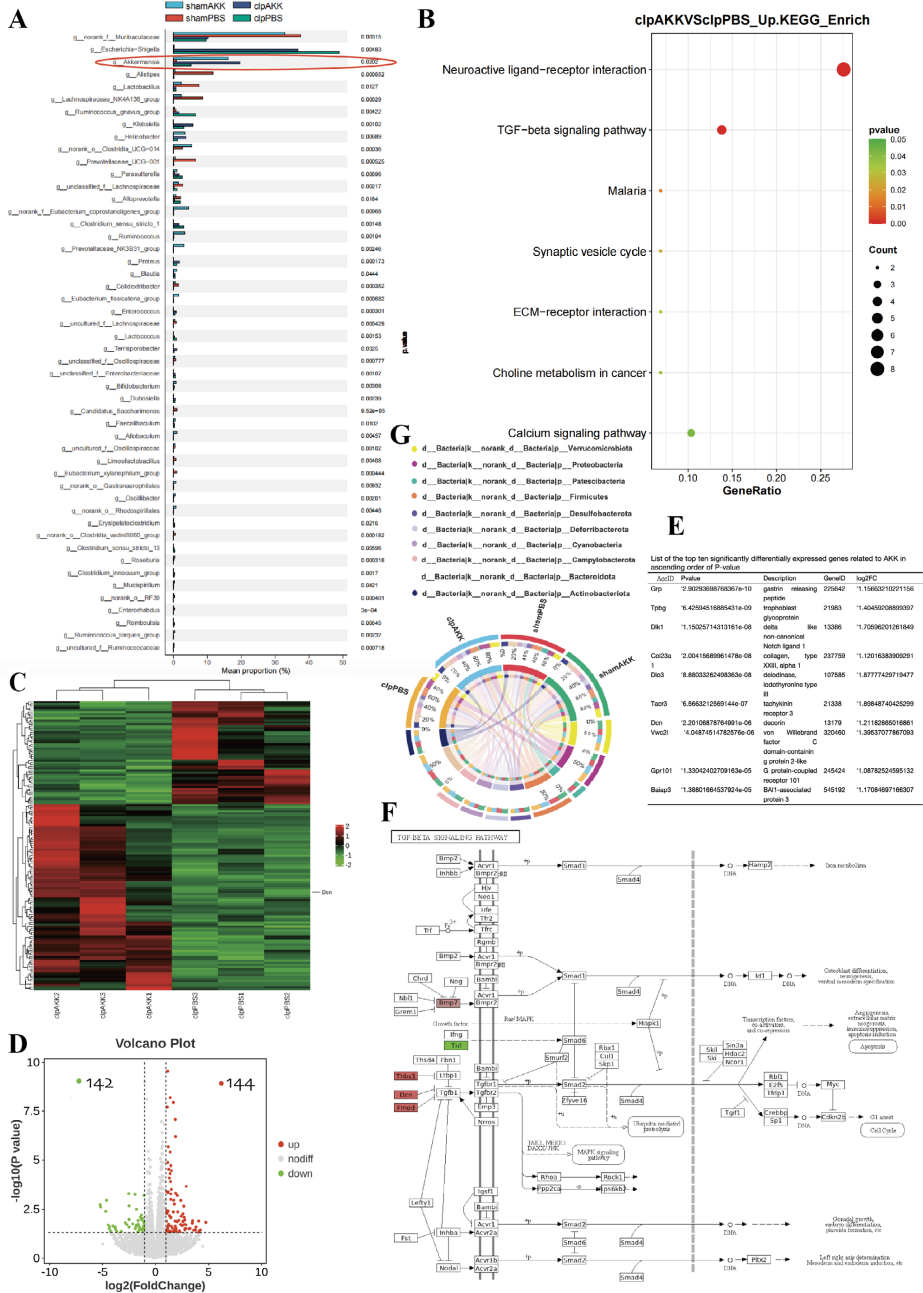
Transcriptomic changes in the mouse hippocampus after AKK administration. (A) The mean abundance of different genera in the genus in Sham + PBS, Sham + AKK, CLP + PBS, CLP + AKK groups (*n* = 6). (B) Top 20 KEGG pathways for the upregulated DEGs. (C) Heatmap of DEGs with a log2FC threshold > 1 and a *p* value < 0.05 (*n* = 3); the DCN gene is indicated. (D) Volcano plots comparing gene expression in the hippocampi of CLP model mice pretreated with AKK or PBS; 24 h of sepsis induction, 144 genes were upregulated (log2FC > 1, Padj < 0.05), and 142 genes were downregulated (log2FC < −1, Padj < 0.05) (*n* = 3 per group). (E) Based on the differential analysis of gene expression in CLP mice after AKK administration, the top 10 genes were selected by sorting in ascending order of *p*‐value and plotted in a table. (F) Diagram of the TGF‐β signaling pathway. DCN is an endogenous inhibitor of this pathway. (G) Relative abundance of microbes at the domain level in the four groups.

To investigate the effects of AKK on gut microbiota in SAE, we performed 16S rRNA sequencing of fecal samples collected 24 h after surgery from sham + PBS, sham + AKK, CLP, and CLP + AKK groups (*n* = 6). At the phylum level, *Verrucomicrobiota* abundance was markedly reduced in groups not receiving AKK, consistent with the taxonomic classification of AKK within this phylum (Figure 3G). At the species level, probiotic abundance was significantly higher in the sham + AKK and CLP + AKK groups compared with controls (Figure 3A). Microbial diversity analysis revealed distinct alterations among groups. Compared with sham + PBS mice, the CLP + PBS group displayed 73 unique microbial species, while sham + PBS mice harbored 225 unique species, with 220 shared species (Figure S1A). Relative to CLP + PBS mice, the CLP + AKK group exhibited 67 unique microbial species, compared with 40 unique species in CLP + PBS mice, with 253 species shared between the two groups (Figure S1B). Alpha diversity analysis using the Chao1, observed, Ace, Shannon, Simpson, and Pielou indices demonstrated significantly reduced microbial diversity in the CLP group compared with sham controls (*p* < 0.01). Notably, diversity in CLP + AKK mice significantly differed from that of CLP mice, with greater intergroup than intragroup variation (Figure S1C,D). Beta diversity analysis using unweighted UniFrac and PCoA further indicated marked differences in community composition between CLP and sham groups (*p* < 0.01). However, no significant compositional differences were observed between CLP and CLP + AKK groups, suggesting that AKK pretreatment did not reverse global community shifts induced by CLP (Figure S1E). At the order level, *Enterobacter* abundance was increased and Bacteroidales abundance was decreased in the CLP group compared with sham. AKK administration increased *Verrucomicrobiales* abundance but had limited effects on other bacterial communities (Figure S1F). To further identify taxa differentially abundant between septic mice with or without AKK pretreatment, LEfSe analysis was performed. Harmful taxa such as *Gammaproteobacteria* and *Escherichia‐Shigella* were enriched in CLP mice compared with sham, but their abundance was reduced after AKK treatment. Conversely, Helicobacter and *Campylobacter* levels were increased in AKK‐pretreated septic mice. Most beneficial genera, including *Prevotella* and *Bifidobacterium*, did not differ significantly with or without AKK. Importantly, AKK administration also increased the abundance of potentially harmful bacteria such as Staphylococcus, suggesting that while AKK modulates certain pathogenic taxa, early supplementation may not restore overall probiotic abundance and may increase susceptibility to some opportunistic infections (Figure S1G–I).

To further explore the molecular targets of AKK, we performed transcriptome sequencing of hippocampal tissue and visualized the results using a volcano plot. Distinct gene expression differences were observed between CLP mice with or without AKK pretreatment, with 144 genes significantly upregulated and 142 genes significantly downregulated (Figure 3D). Heatmap analysis further illustrated clear clustering patterns and differential expression profiles across groups (Figure 3C). Functional enrichment analyses were then conducted to elucidate the biological pathways associated with these DEGs. KEGG pathway and GO enrichment analyses identified the top 20 significantly enriched pathways in AKK‐pretreated CLP mice (Figure S2). Pathways enriched in the upregulated gene set are shown in Figure 3B. KEGG results indicated that many of these genes were linked to TGF‐β and calcium signaling pathways. Interestingly, inhibitors of TGF‐β signaling—including Fmod, Thbs1, and Dcn—were themselves upregulated (Figure 3F), with Dcn ranking among the top 10 genes by *p*‐value screening (Figure 3E). Given our longstanding research on Dcn in autophagy, the identification of this gene among the most significantly altered DEGs highlights it as a potential key target for studying disease mechanisms. We therefore further investigated the interactions among AKK, Dcn, and autophagy to clarify the mechanistic role of AKK in SAE. Collectively, these findings suggest that AKK supplementation influences hippocampal gene expression and may modulate pathways relevant to SAE pathogenesis through intestinal regulation.

### 3.3 AKK Modulates DCN‐Mediated Autophagy and Apoptosis in Hippocampal Neurons

**FIGURE 4:**
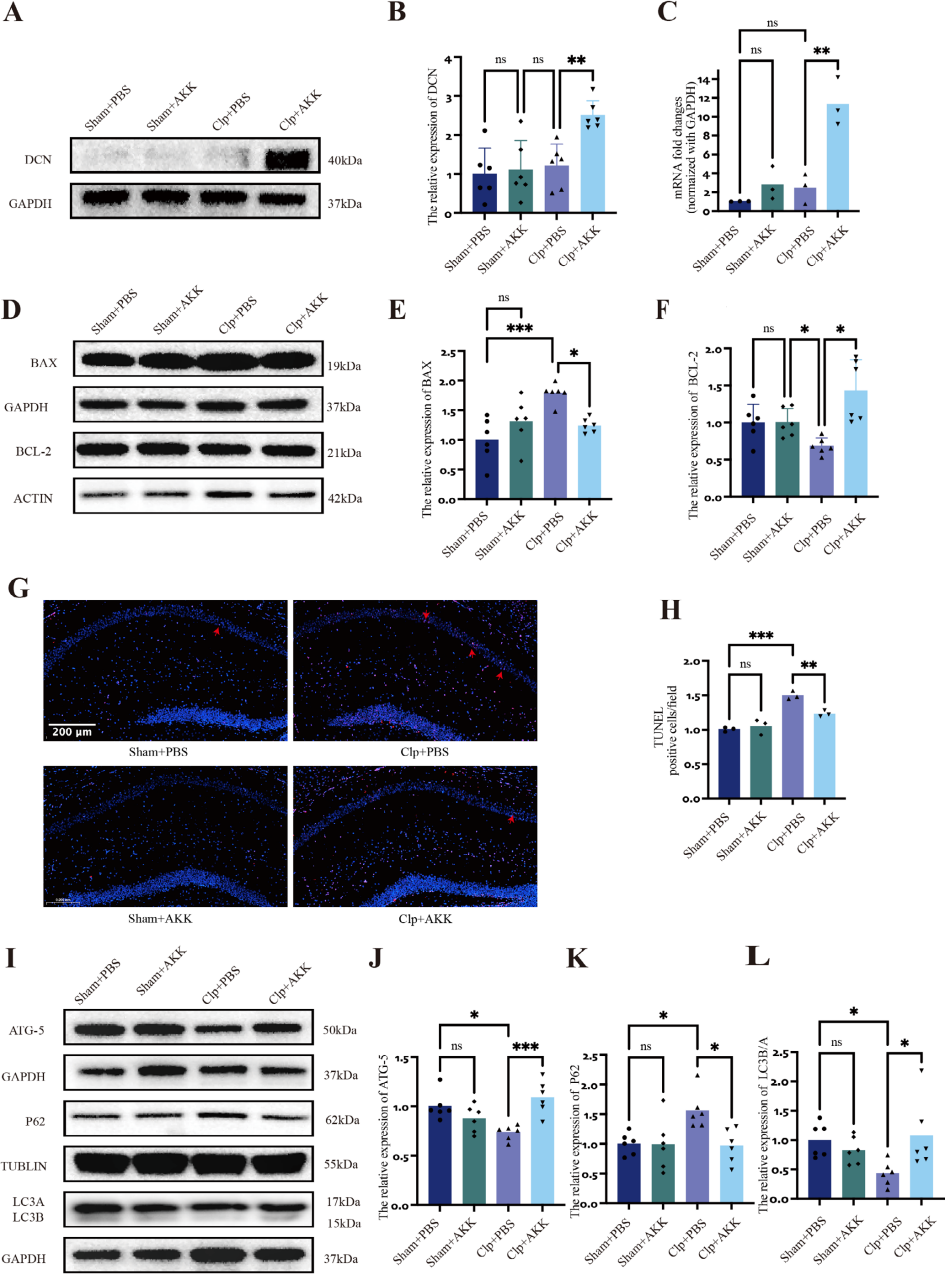
AKK can reduce hippocampal inflammation and increase autophagy. (A) Western blot analysis of DCN expression after CLP, AKK treatment, or both (*n* = 6). (B) Western blot analysis and quantitative analysis of total protein expression. (C) mRNA levels of DCN in the hippocampi of mice pretreated with the control or live AKK (*n* = 3). (D) Western blot analysis and quantitative analysis of BAX and BCL‐2 protein levels in hippocampal tissue (*n* = 6). (E, F) Western blot analysis and quantitative analysis of BAX and BCL‐2 protein expression. (G) TUNEL staining was used to measure the level of apoptosis in hippocampal neurons (scale: 200 μm, magnification: ×20) (*n* = 3). (H) Quantitative analysis of TUNEL staining. (I) Western blot analysis and quantitative analysis of ATG5, p62, and LC3 protein levels in hippocampal tissue (*n* = 6). (J–L) Western blot analysis and quantitative analysis of ATG5, p62, and LC3 protein expression. The data are presented as the means ± SD. *p < 0.05, **p < 0.01, ***p < 0.001, and ****p < 0.0001.

**FIGURE 5:**
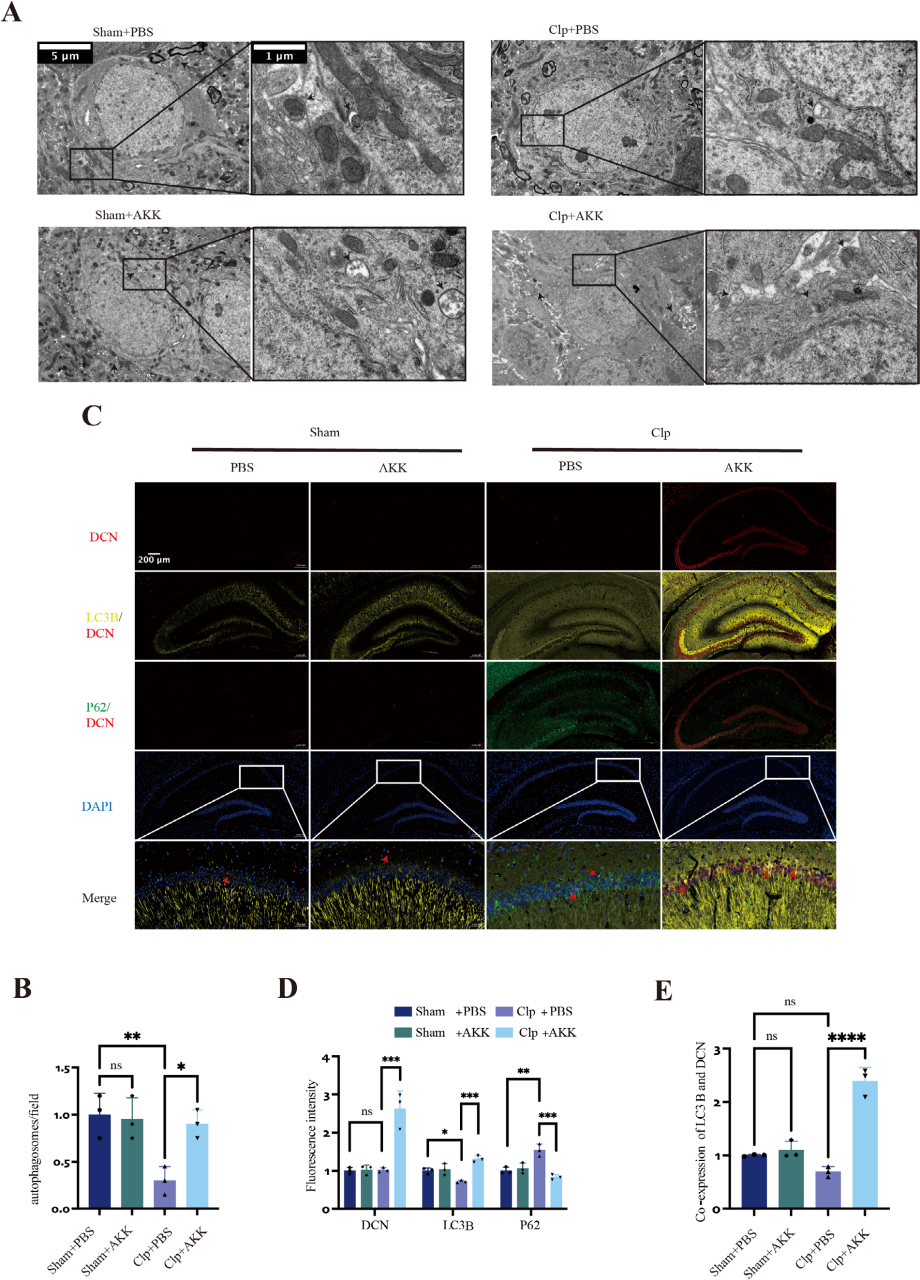
AKK may increase autophagosome formation by upregulating DCN expression. (A) Ultrastructural analysis by transmission electron microscopy showing autophagosomes in the hippocampus of the mice (scale: 5 μm/1 μm, magnification: ×8/×40) (*n* = 3). (B) Statistical analysis of the number of autophagosomes. (C) Co localization of LC3, P62, and DCN among the four groups(scale: 200 μm, magnification: ×10) (*n* = 3). (D, E) Quantitative analysis of fluorescence intensity. The images are representative of at least three independent experiments. The data are presented as the means ± SD. *p < 0.05, **p < 0.01, ***p < 0.001, and ****p < 0.0001.

We next investigated the mechanisms underlying CA1 neuronal injury in SAE and the therapeutic effects of AKK. Prior evidence suggests an association between DCN and autophagy, whereby increased DCN expression can enhance autophagy in multiple tissues and reduce apoptosis. Consistent with this, transcriptomic analysis revealed that in CLP mice pretreated with AKK, both DCN mRNA and protein expression were significantly elevated in the hippocampus (*F*(3,8) = 8.852, *p* = 0.0011; WB, *F*(3,20) = 5.268, *p* = 0.0067) (Figure 4A–C). To determine whether autophagy contributes to the protective effects of AKK, we examined apoptosis‐ and autophagy‐related proteins. The Bcl‐2 family, including the anti‐apoptotic protein BCL‐2 and the pro‐apoptotic protein BAX, plays a central role in mitochondria‐dependent apoptotic regulation. CLP markedly reduced hippocampal BCL‐2 expression (*F*(3,20) = 3.523, *p* = 0.0400) while increasing BAX expression (*F*(3,20) = 6.693, *p* = 0.0007). In contrast, AKK pretreatment restored BCL‐2 levels (*F*(3,20) = 5.707, *p* = 0.0260) and suppressed BAX expression (*F*(3,20) = 4.655, *p* = 0.0176) (Figure 4D–F). Similarly, TUNEL staining demonstrated a significant increase in apoptotic cells in the hippocampal CA1 region of CLP mice, which was attenuated by AKK treatment (*F*(3,8) = 12.28, *p* = 0.0001; *F*(3,8) = 6.622, *p* = 0.0069) (Figure 4G,H). We then assessed autophagy‐related proteins. ATG5 is indispensable for autophagosome elongation, LC3‐II marks autophagosome formation, and P62 acts as an adaptor for substrate delivery to LC3‐II, with its accumulation reflecting impaired autophagic flux [22]. In the CLP group, hippocampal ATG5 and LC3 expression were reduced, while P62 levels were elevated (ATG5, *F*(3,20) = 4.877, *p* = 0.0125; LC3, *F*(3,20) = 2.980, *p* = 0.0173; P62, *F*(3,20) = 4.877, *p* = 0.0293). AKK pretreatment reversed these changes, significantly increasing ATG5 (*F*(3,20) = 6.501, *p* = 0.0009) and LC3 (*F*(3,20) = 2.817, *p* = 0.0291) while reducing P62 (*F*(3,20) = 4.577, *p* = 0.0198) (Figure 4I–L). Transmission electron microscopy provided ultrastructural evidence of these findings. CLP mice exhibited a reduced number of hippocampal autophagosomes compared with sham controls (Figure 5A,B), while AKK significantly increased autophagosome numbers (*F*(3,8) = 6.261, *p* = 0.0095; *F*(3,8) = 5.367, *p* = 0.0220). In physiological conditions, autolysosomes (ALs) contain single membranes and electron‐dense degraded material. Notably, CLP mice exhibited few mature ALs, suggesting defective autophagosome clearance. By contrast, AKK pretreatment restored mature AL formation, indicating intact autophagic flux. These data support that AKK confers neuroprotection by simultaneously enhancing autophagy and inhibiting apoptosis in hippocampal neurons. Finally, to verify the involvement of DCN in these processes, we performed dual immunofluorescence staining for LC3B, P62, and DCN (Figure 5C). DCN expression did not differ significantly between CLP and sham groups, though autophagy‐related protein patterns (LC3B, P62) mirrored WB results. Strikingly, AKK pretreatment upregulated DCN, increased LC3B, and reduced P62 in hippocampal neurons (LC3B, *F*(3,8) = 5.078, *p* = 0.0291; P62, *F*(3,8) = 7.734, *p* = 0.0026; LC3B, *F*(3,8) = 10.64, *p* = 0.0003; P62, *F*(3,8) = 10.11, *p* = 0.0004; DCN, *F*(3,8) = 11.11, *p* = 0.0002) (Figure 5D). Colocalization analysis further revealed a marked increase in LC3–DCN overlap in AKK‐treated CLP mice (*F*(3,8) = 18.13, *p* < 0.0001) (Figure 5E).

Together, these results suggest that DCN may act as an effective autophagy inducer, facilitating autophagosome formation and substrate targeting, and thereby promoting protective autophagic flux in SAE.

### 3.4 Disruption of DCN Abolishes the Neuroprotective Effects of AKK in SAE

**FIGURE 6:**
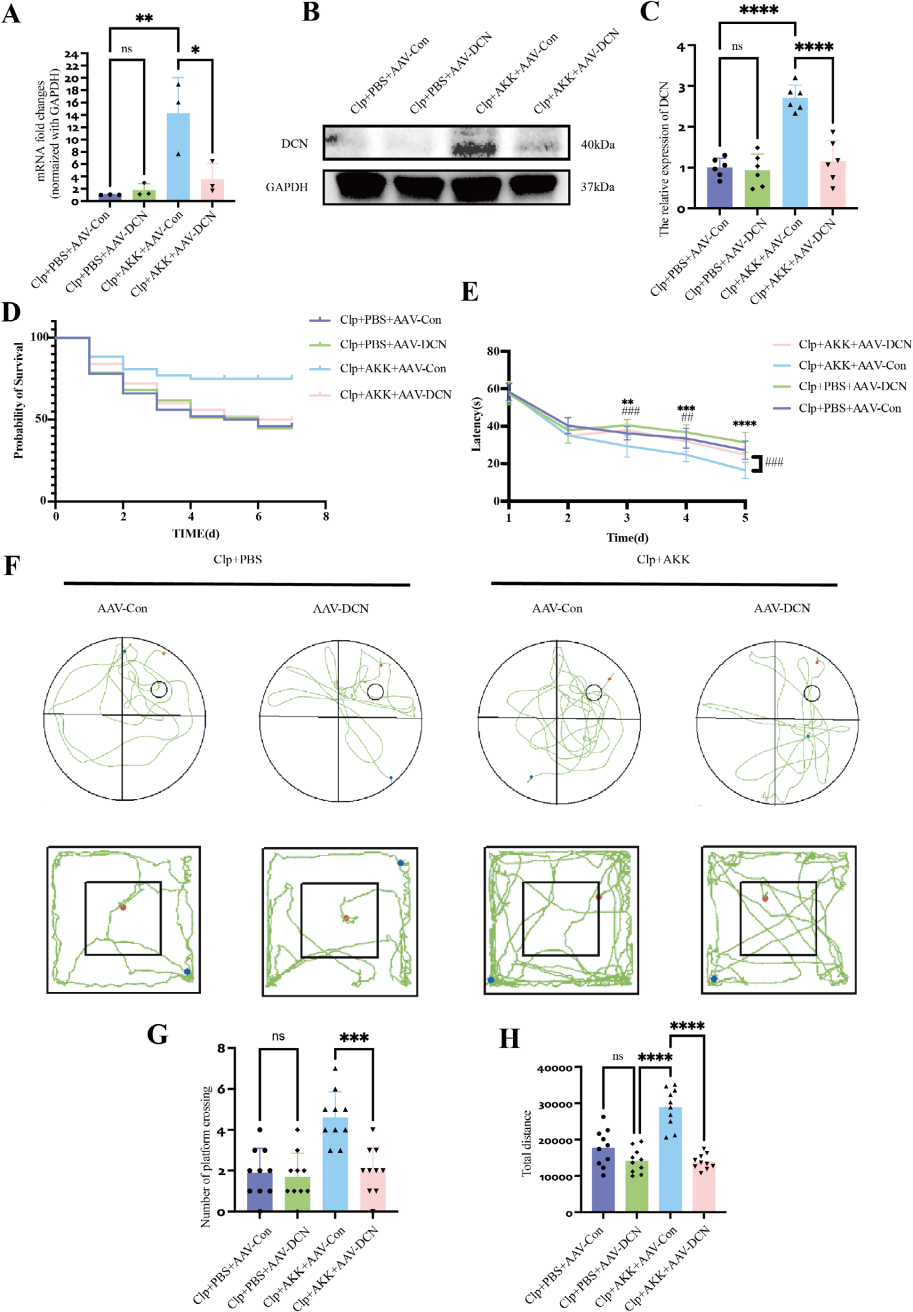
AKK may reduce mortality and alleviate SAE by increasing DCN gene expression. (A) The groups treated with or without the virus to modulate DCN mRNA levels in the hippocampus were pretreated with live AKK or PBS (*n* = 3). (B, C) Western blot analysis of DCN expression and quantification of total protein expression (*n* = 6). (D) Twenty‐one days after virus or PBS was injected into the lateral ventricles of the mice, live AKK or PBS (200 μL each time) was administered for 3 days (once a day, surgery was performed 2 h after the third treatment), followed by CLP or sham surgery. A Kaplan–Meier curve was used to determine the survival rate (*n* = 50 mice per group). (E–H) MWM test and OFT (*n* = 10). (E) Escape latency of the mice during the training phase of the MWM test. (F) Trajectory maps of the mice in the MWM test and OFT. (G) Number of times platform crossings during the postoperative probe phase of the MWM test. (H) The total distance traveled in the OFT by each group of mice. The data are presented as the means ± SD. *p < 0.05, **p < 0.01, ***p < 0.001, and ****p < 0.0001.

To determine whether increased DCN expression mediates the effects of AKK on hippocampal apoptosis and autophagy in SAE, we disrupted DCN expression in vivo by intracerebroventricular injection of AAV‐DCN or AAV‐Control 3 weeks before CLP surgery. Both PCR (*F*(3,8) = 5.721, *p* = 0.0157) and Western blotting (*F*(3,20) = 0.050, *p* < 0.0001) confirmed efficient suppression of DCN expression (Figure 6A–C). Strikingly, DCN inhibition abolished the survival advantage conferred by AKK in CLP mice (Figure 6D). Cognitive testing further supported this effect. In the Morris water maze, no significant differences in learning performance were observed between AKK‐treated mice with viral interference and CLP mice without probiotic treatment. In contrast, AKK significantly improved cognitive and behavioral performance in CLP mice, which was abolished by DCN inhibition. Latency times over five consecutive training days were prolonged in virus‐injected mice (Day 3, *F*(3,36) = 5.733, *p* = 0.0004; Day 4, *F*(3,36) = 4.690, *p* = 0.0060; Day 5, *F*(3,36) = 5.526, *p* = 0.0008; probe test, *F*(3,36) = 6.881, *p* = 0.0001; *F*(3,36) = 11.62, *p* < 0.0001) (Figure 6E). Consistently, trajectory analyses showed that viral interference markedly reduced the number of platform crossings (*F*(3,36) = 13.19, *p* < 0.0001) (Figure 6F,G). In the open‐field test, total locomotor distance was significantly reduced in virus‐injected mice compared with AKK‐treated controls (*F*(3,36) = 28.27, *p* < 0.0001) (Figure 6F,H). We next assessed hippocampal pathology and inflammation following DCN suppression. H&E staining revealed that inhibition of DCN expression aggravated CA1 neuronal injury, with more loosely arranged neurons and an increased proportion of deeply stained cells in the CLP + AKK + AAV‐DCN group compared with the CLP + AKK + AAV‐Control group (Figure S3A). Notably, hippocampal IL‐1β and TNF‐α levels were not significantly altered (Figure S3B,C), suggesting that the effects were not driven by changes in local inflammatory cytokines. Nissl staining further confirmed neuronal vulnerability: virus‐pretreated CLP mice displayed increased neuronal loss, vacuolization, and reduced numbers of Nissl‐positive neurons relative to AKK‐treated CLP mice (*F*(3,8) = 5.825, *p* = 0.0142) (Figure 3D,E). Together, these results demonstrate that suppression of DCN abolishes the protective effects of AKK against hippocampal damage and cognitive impairment in SAE, supporting a central role for DCN in mediating the neuroprotective mechanism of AKK.

### 3.5 DCN Contributes to the Preventive and Therapeutic Effects of AKK on SAE by Increasing Autophagy

**FIGURE 7:**
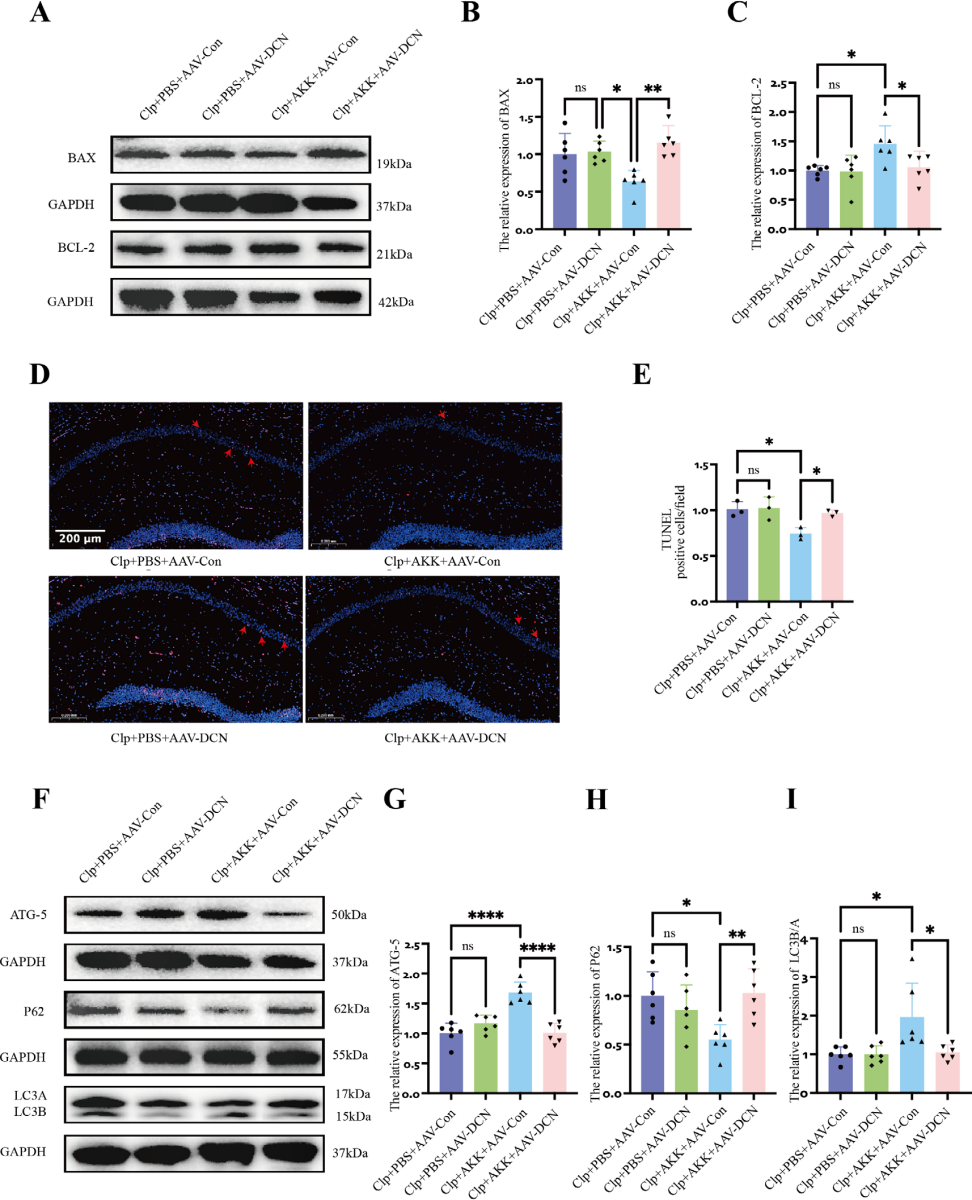
Inhibition of DCN expression prevents AKK from exerting positive effects by increasing autophagy. (A–C) Western blot analysis and quantitative analysis of BAX and BCL2 protein levels in hippocampal tissue (*n* = 6). (D, E) TUNEL staining was used to measure the level of apoptosis in hippocampal neurons (*n* = 3) and quantitative analysis of TUNEL staining (scale: 200 μm, magnification: ×20) (*n* = 3). (F–I) Western blot analysis and quantitative analysis of ATG5, p62, and LC3 protein levels in hippocampal tissue (*n* = 6). The data are presented as the means ± SD. *p < 0.05, **p < 0.01, ***p < 0.001, and ****p < 0.0001.

**FIGURE 8:**
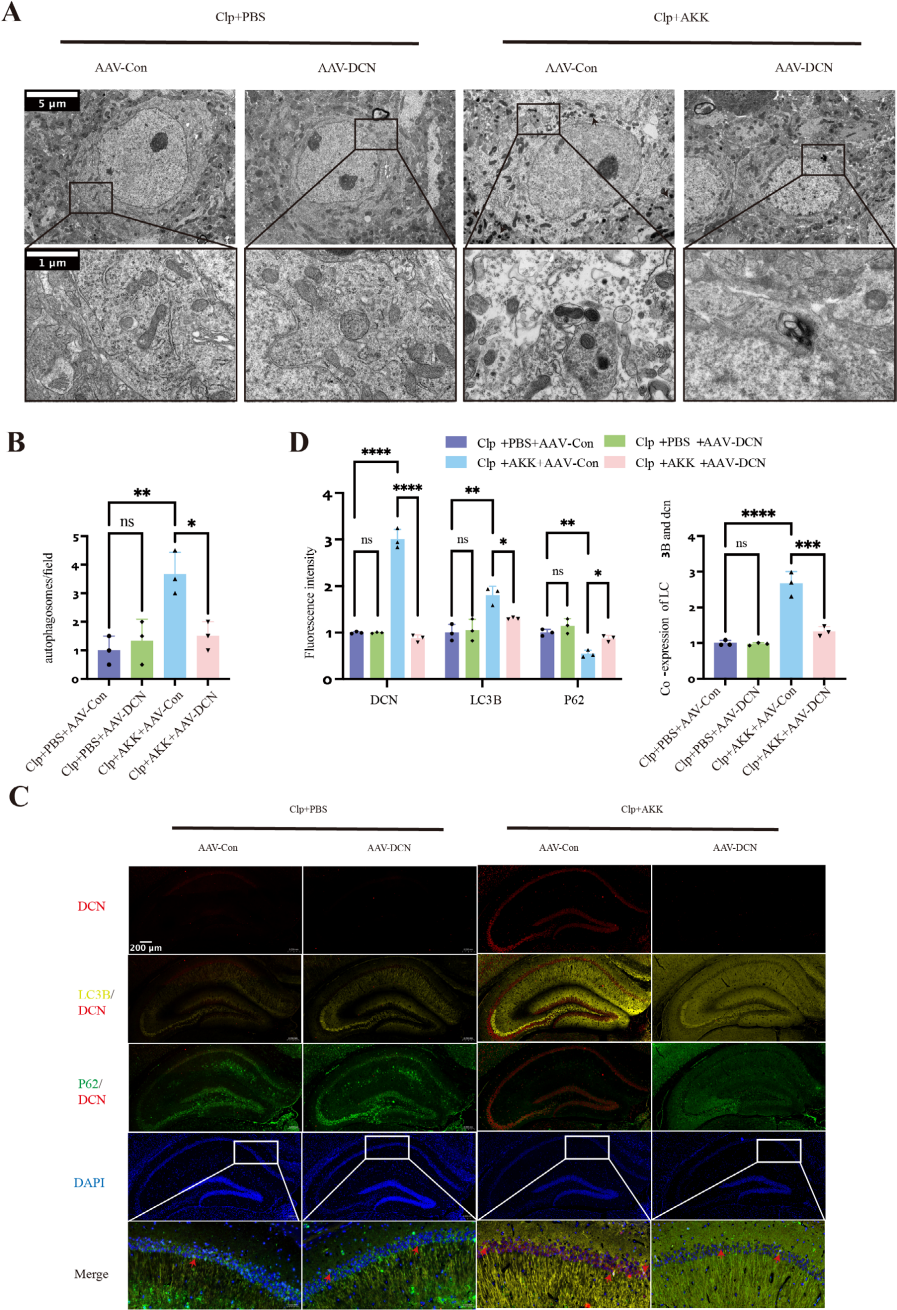
AKK increases DCN expression to elevate autophagosome formation in hippocampal tissue. (A) Ultrastructural analysis by transmission electron microscopy showing autophagosomes in the hippocampi of the mice (scale: 5 μm/1 μm, magnification: ×8/×40) (*n* = 3). (B) Statistical analysis of the number of autophagosomes. (C) Co‐localization of LC3, P62, and DCN among the four groups. The images are representative at least three independent experiments(scale: 200 μm, magnification: ×10) (*n* = 3). (D) Quantitative analysis of fluorescence intensity. The data are presented as the means ± SD. *p < 0.05, **p < 0.01, ***p < 0.001, and ****p < 0.0001.

Next, we examined the effects of DCN knockdown on hippocampal apoptosis‐ and autophagy‐related proteins in the different groups. In virus‐injected mice, expression of the pro‐apoptotic protein BAX increased, while BCL‐2 expression decreased (*F*(3,20) = 6.412, *p* = 0.0011; *F*(3,20) = 4.062, *p* = 0.0430) (Figure 7A–C). Consistently, TUNEL staining revealed a significant increase in apoptotic cells in the CA1 region (*F*(3,8) = 4.585, *p* = 0.0473) (Figure 7D,E). These findings indicate that suppression of DCN eliminates the anti‐apoptotic effect of AKK on hippocampal neurons. We then analyzed autophagy markers. Compared with CLP + AKK + AAV‐Control mice, CLP + AKK + AAV‐DCN mice showed P62 accumulation, reduced ATG‐5 expression, and decreased LC3 levels (P62, *F*(3,20) = 5.037, *p* = 0.0097; ATG‐5, *F*(3,20) = 10.06, *p* < 0.0001; LC3, *F*(3,20) = 2.817, *p* = 0.0291) (Figure 7F–I). Transmission electron microscopy further confirmed a reduction in hippocampal autophagosomes following viral interference (*F*(3,8) = 5.814, *p* = 0.0144) (Figure 8A,B). Mature autophagosomes were rarely observed, suggesting that DCN is essential for complete autophagic clearance and that the protective effects of AKK depend on both enhanced autophagy and reduced neuronal apoptosis. To visualize the impact of DCN on autophagy more directly, we performed co‐localization analyses of key autophagy proteins in hippocampal slices. Relative to CLP + AKK + AAV‐Control mice, CLP + AKK + AAV‐DCN mice exhibited significantly reduced DCN, LC3, and P62 expression (LC3, *F*(3,8) = 4.920, *p* = 0.0340; P62, *F*(3,8) = 5.680, *p* = 0.0163; DCN, *F*(3,8) = 32.70, *p* < 0.0001), as well as decreased LC3–DCN co‐localization (*F*(3,8) = 12.29, *p* = 0.0001) (Figure 8C,D). These results indicate that DCN enhances autophagic degradation (lysosomal function) in neurons with inherent defects, thereby reducing autophagic “blockage.”

## 4 Discussion

After sepsis, the intestinal barrier is highly vulnerable to damage, which can accelerate disease progression. Similarly, CLP disrupts the gut microbiota, as evidenced by marked alterations in microbial composition and species abundance [23]. The gut microbiota is a major upstream regulator of sepsis, and increasing evidence suggests that the gut–brain axis plays an important role in the pathogenesis of SAE, indicating that microbial composition influences susceptibility to SAE [24]. *Akkermansia muciniphila* (AKK) is a next‐generation probiotic with proven protective effects on the intestinal barrier in multiple metabolic disease models [25]. However, its role in the gut–brain axis remains largely unexplored, and its ability to regulate gene function warrants further investigation to determine whether it can be used to treat SAE.

In our study, we uncovered a potential association between AKK and the progression of SAE in mice. Notably, AKK not only reduced sepsis‐induced hippocampal inflammation but also upregulated DCN expression, enhanced autophagy in hippocampal CA1 neurons, reduced apoptosis, and ultimately prevented SAE. We hypothesize that certain neuronal populations, such as hippocampal CA1 neurons and cortical neurons, may be particularly sensitive to these effects. Their high energy demand and vulnerability to stress make them more dependent on autophagy for homeostasis, thereby eliciting a stronger response to the metabolic regulatory signals of AKK [21]. Previous studies have also shown that AKK strengthens the intestinal mucosal barrier and reduces the translocation of LPS into the bloodstream [26]. Lower circulating LPS levels reduce activation of Toll‐like receptor 4 (TLR4), thereby suppressing the downstream NF‐κB inflammatory pathway and alleviating neuroinflammation [15]. Consistent with this, transcriptomic analysis in our study revealed that this pathway was downregulated in the hippocampus of AKK‐treated mice (Figure S2). Taken together, these findings suggest that inhibition of the TLR4/NF‐κB signaling pathway may underlie the observed reduction in hippocampal inflammatory factors in SAE mice following AKK treatment. We further sought to determine whether DCN contributes to the protective autophagic response in SAE. DCN expression was found to be low in the normal mouse brain, and silencing DCN reduced autophagic flux while abolishing the beneficial effects of AKK on hippocampal tissue. These findings indicate that DCN protects brain tissue in an autophagy‐dependent manner. Although memory impairment is the hallmark symptom of SAE, the condition encompasses a broader syndrome of global brain dysfunction [27]. Thus, focusing solely on the CA1 region may overlook non‐memory‐related but equally disabling symptoms associated with SAE [20]. Future research should adopt a more holistic approach to SAE pathophysiology.

The marked reduction of *Akkermansia muciniphila* (AKK) in the gut of sepsis patients provides a strong rationale for exploring it as a gut–brain axis–targeted therapy for SAE. As a next‐generation probiotic, AKK has attracted considerable attention due to its ability to strengthen the intestinal barrier, reduce systemic inflammation, and produce beneficial metabolites. Representing more than 1% of the healthy human gut microbiota and already available commercially [28], AKK offers promising translational potential. Possible strategies include oral administration of live or inactivated AKK, as well as prebiotics that promote its growth [29]. Such interventions could help restore the sepsis‐compromised intestinal barrier, limit bacterial translocation and systemic inflammation, and thereby indirectly protect the brain and improve long‐term cognitive outcomes. Beyond barrier protection, AKK exerts systemic effects through both bioactive products and direct gene regulation [29]. Its primary metabolites, acetic acid and propionic acid, are short‐chain fatty acids (SCFAs) that can cross the blood–brain barrier and activate neuronal G protein–coupled receptors, influencing neuronal function and gene expression through epigenetic regulation [30, 31]. Our study demonstrates for the first time that in severe sepsis, AKK can directly regulate hippocampal gene expression, upregulate DCN, enhance autophagy, and reduce neuronal apoptosis, highlighting a potential mechanism for its neuroprotective effects. However, the molecular pathways linking AKK to DCN remain unclear. Because DCN was identified through omics screening, no evidence currently describes the transcriptional or signaling events involved. We speculate that regulation is complex and may involve multiple overlapping mechanisms. Moreover, AKK is unlikely to act in isolation; other bacterial taxa and metabolites may also contribute to sepsis pathophysiology [32]. Future work should delineate the signaling cascades by which AKK regulates DCN and identify additional bioactive metabolites and functional genes critical for SAE progression. Despite these uncertainties, the therapeutic promise of AKK is considerable. Translation into clinical practice will require overcoming challenges such as large‐scale GMP production, optimization of dosing regimens, and safety validation in vulnerable patient populations. Nonetheless, interventions targeting AKK provide a compelling, noninvasive, and innovative approach to SAE—a condition for which effective therapies remain urgently needed.

Emerging evidence suggests that autophagy functions as a double‐edged sword under both physiological and pathological conditions, exerting protective or detrimental effects depending on its activation level [33]. Mild activation below a critical threshold facilitates the clearance of damaged cellular components and supports cell survival [34]. In contrast, excessive activation can trigger autophagic cell death, whereas insufficient activation compromises cellular homeostasis. Notably, several studies have reported reduced autophagy in sepsis models [35]. To date, only limited research has investigated autophagy in SAE [16], and its pathological dynamics remain poorly defined. In the early stages of SAE, autophagy activation may act as a compensatory protective mechanism. However, as sepsis progresses, severe inflammation and oxidative stress may overwhelm the autophagic machinery, leading to impaired autophagic flux—manifested by p62 accumulation—and diminished protective capacity [36]. This impaired autophagy exacerbates neuronal injury through a vicious cycle involving neuroinflammation, mitochondrial dysfunction, and neuronal death [37]. Consistent with this, our findings demonstrate that hippocampal autophagy is suppressed in SAE under moderate‐to‐severe sepsis conditions, and that appropriately enhancing autophagy can ameliorate cognitive impairment and mitigate tissue damage. Over the past decade, DCN has been extensively studied as a tumor suppressor gene [38], but increasing attention has also focused on its roles in inflammation and autophagy [39]. Autophagy activation has been shown to regulate DCN‐mediated proteoglycan expression [40], and DCN itself can induce autophagy across multiple cell types. However, because autophagy is modulated by diverse signaling pathways, DCN has not yet been widely explored as a therapeutic target [41]. As a natural inhibitor of TGF‐β, DCN is often upregulated in response to activation of this pathway. Our transcriptomic data revealed enhanced TGF‐β signaling in the hippocampus of AKK‐treated mice, suggesting that increased DCN expression may be directly linked to this pathway. Nevertheless, the precise role of TGF‐β signaling in sepsis remains unclear [42]. Beyond this, accumulating evidence highlights the AMPK/mTOR axis as a central regulator of autophagy in sepsis [43]. DCN has been reported to activate AMPK (thereby promoting autophagy) and inhibit mTOR (relieving its suppression of autophagy and potentially reducing apoptosis). In addition, DCN facilitates the dissociation of Bcl‐2 from Beclin‐1, thereby releasing Beclin‐1 to initiate autophagy while simultaneously attenuating apoptosis [44, 45]. Taken together, these findings underscore the need for further investigation into how AKK regulates hippocampal DCN expression to modulate autophagy and apoptosis, ultimately influencing cognitive outcomes in SAE.

In summary, we provide new evidence for host–microbiome interactions in the context of sepsis‐related brain injury. By demonstrating that live AKK regulates hippocampal DCN expression, we expand the understanding of how probiotics influence neuropathology in SAE. If DCN proves protective against SAE, particularly regarding long‐term sepsis prognosis, it may represent a promising therapeutic target.

## Author Contributions

Bingqing Gong and Feixiang Li designed the study and wrote the protocol. Bingqing Gong and Yuanyuan Bai managed the literature searches and analyses. Guo Yang and Bingqing Gong undertook the statistical analysis. Zhang Rui and Feixiang Li are responsible for data analysis. Yonghao Yu provided funding. Beibei Dong provided the original concept and funding. Bingqing Gong and Feixiang Li have the same contribution to this article. All authors have reviewed the manuscript.

## Consent

All authors contributed to and have approved the final manuscript.

## Conflicts of Interest

The authors declare no conflicts of interest.

## Supporting information


**Figures S1–S3:** cns70642‐sup‐0001‐FigureS1‐S3.docx.

## Data Availability

The data that support the findings of this study are openly available in Sequence Read Archive (SRA) at https://submit.ncbi.nlm.nih.gov/subs/sra/, reference number SUB15655642.
