# Supplementary material for: Decorin Is a Newly Discovered Target of Akkermansia muciniphila in the Treatment of Sepsis‐Associated Encephalopathy
Source: CNS Neurosci Ther. 2025 Nov 4;31(11):e70642. doi: 10.1111/cns.70642 (PMC12584041; doi:10.1111/cns.70642)
Supplement: Supplementary file 1 — Figures S1–S3: cns70642‐sup‐0001‐FigureS1‐S3.docx. [file CNS-31-e70642-s001.docx]

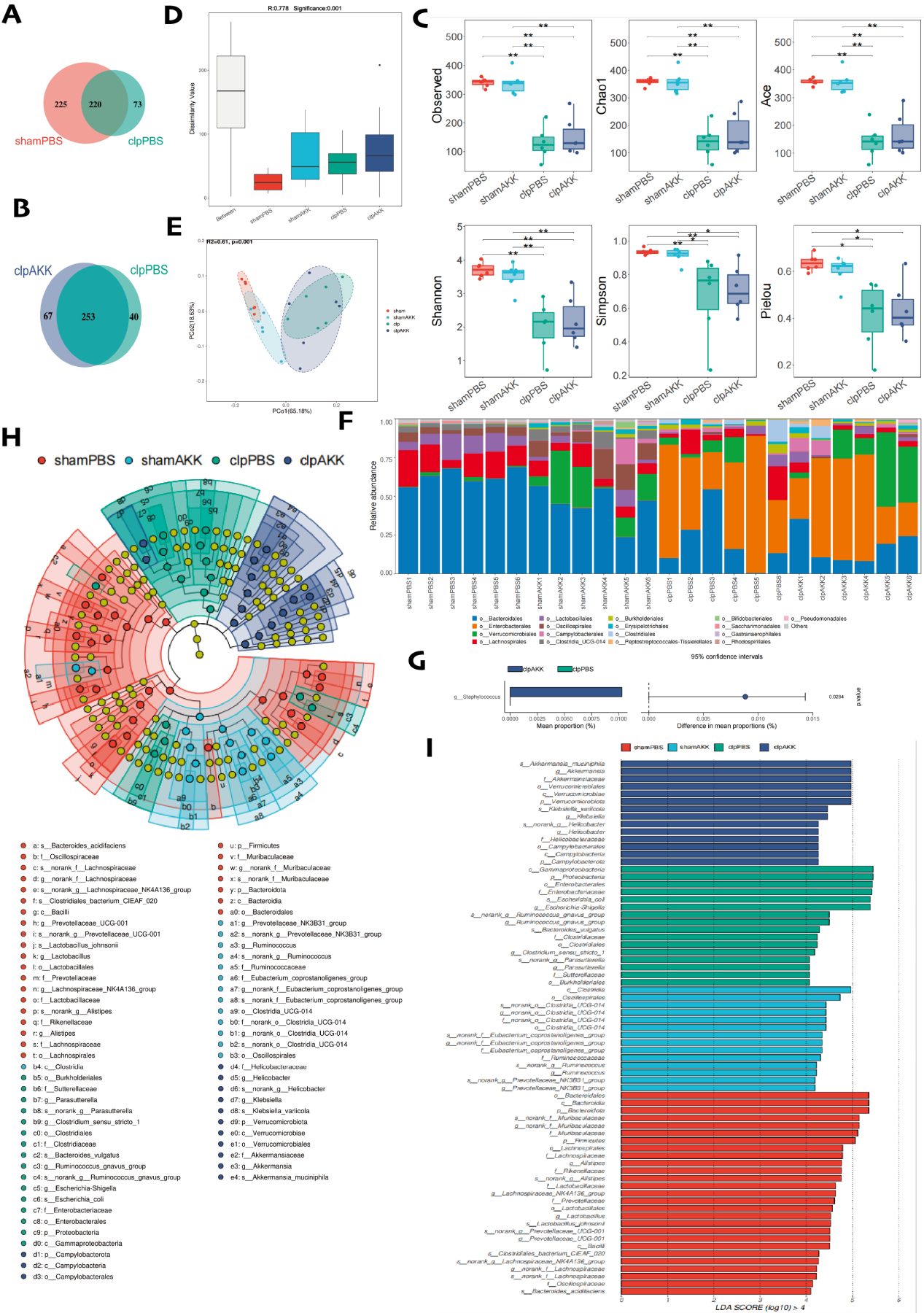


Supplementary Figure 1

Early administration of AKK by gavage does not reverse the disruption of the gut microbiota caused by CLP.

(A, B) Venn diagram showing the number of common and unique OTUs among the different groups. (C) One-way ANOVA revealed differences in the Shannon, Observed, Chao1, Ace, Simpson, and Pielou indices between septic mice (n=6) and normal mice (n=6), but there was no significant difference in these indices after CLP and AKK administration. The data are presented as the median and quartiles. *p < 0.05, ** p<0.01.（D) There were significant differences in microbial community structure among the four groups (ANOSIM, R=0.775, p=0.001) (E) PCoA of the Bray–Curtis distance among the sham, sham+AKK, sepsis and sepsis+AKK groups. Each point represents a sample, and the colours represent the different groups. The results of ANOSIM of diversity indices among the groups are shown above the plots. (F) Average relative abundance of the main orders among the four groups. (G) The changes in gut microbiota composition between the two groups were statistically significant at the genus level. (H, I) LEfSe analysis of the gut microbiota. (I) Linear discriminant analysis (LDA) scores (phylum to species level) among the four groups. Only taxa with an LDA value > 4 are presented. (H) Classification branching diagram obtained from LEfSe analysis of the 16S sequences. The circles indicate phylogenetic levels, and the species with no significant difference in abundance are shown in yellow. The diameter and colour of each circle represent the abundance and enterotype, respectively. *p < 0.05, **p < 0.01, ***p < 0.001.


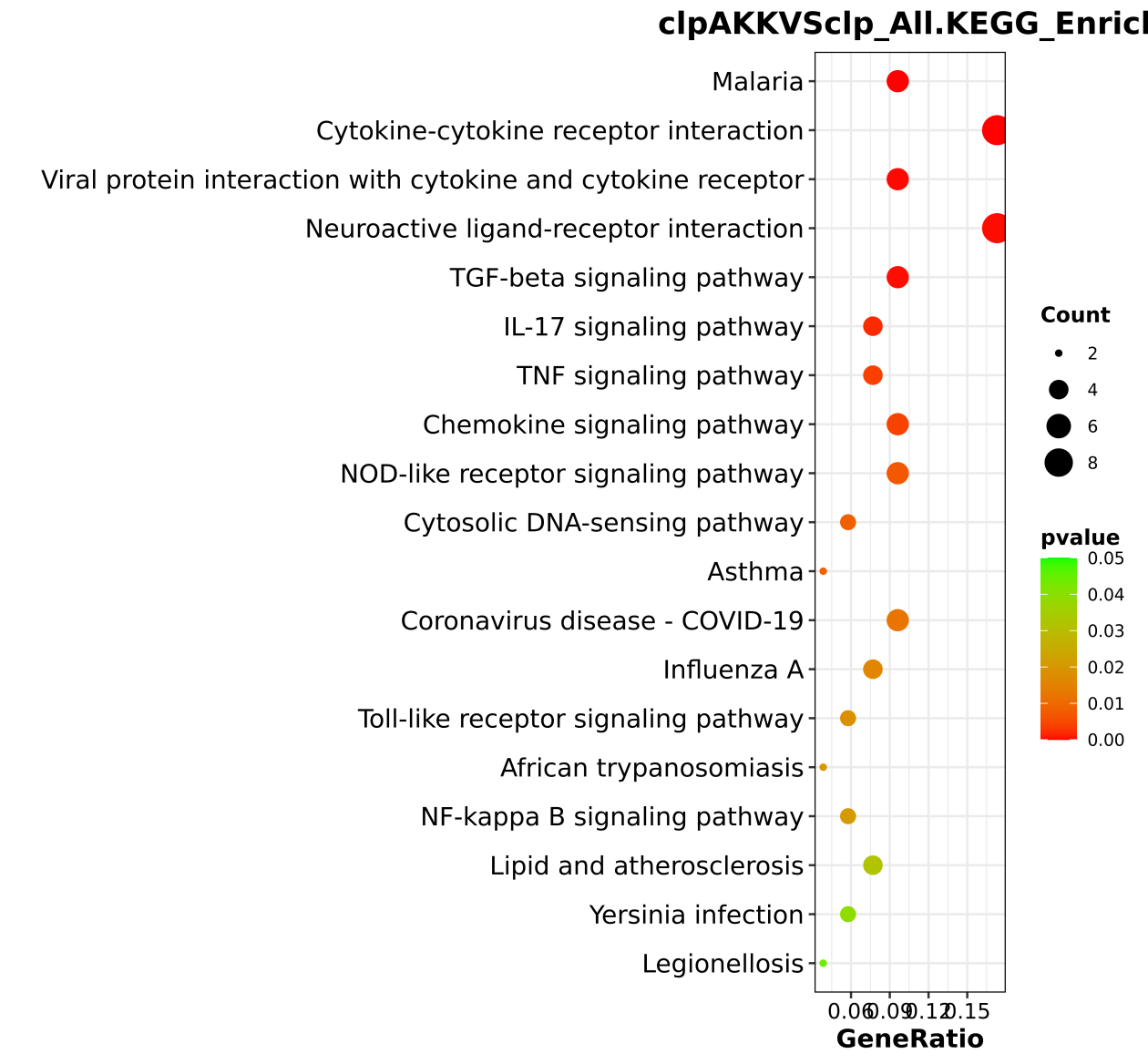


Supplementary Figure 2

Regulation of AKK on different signaling pathways in the hippocampus of CLP mice

Top 20 KEGG pathways for the DEGs.


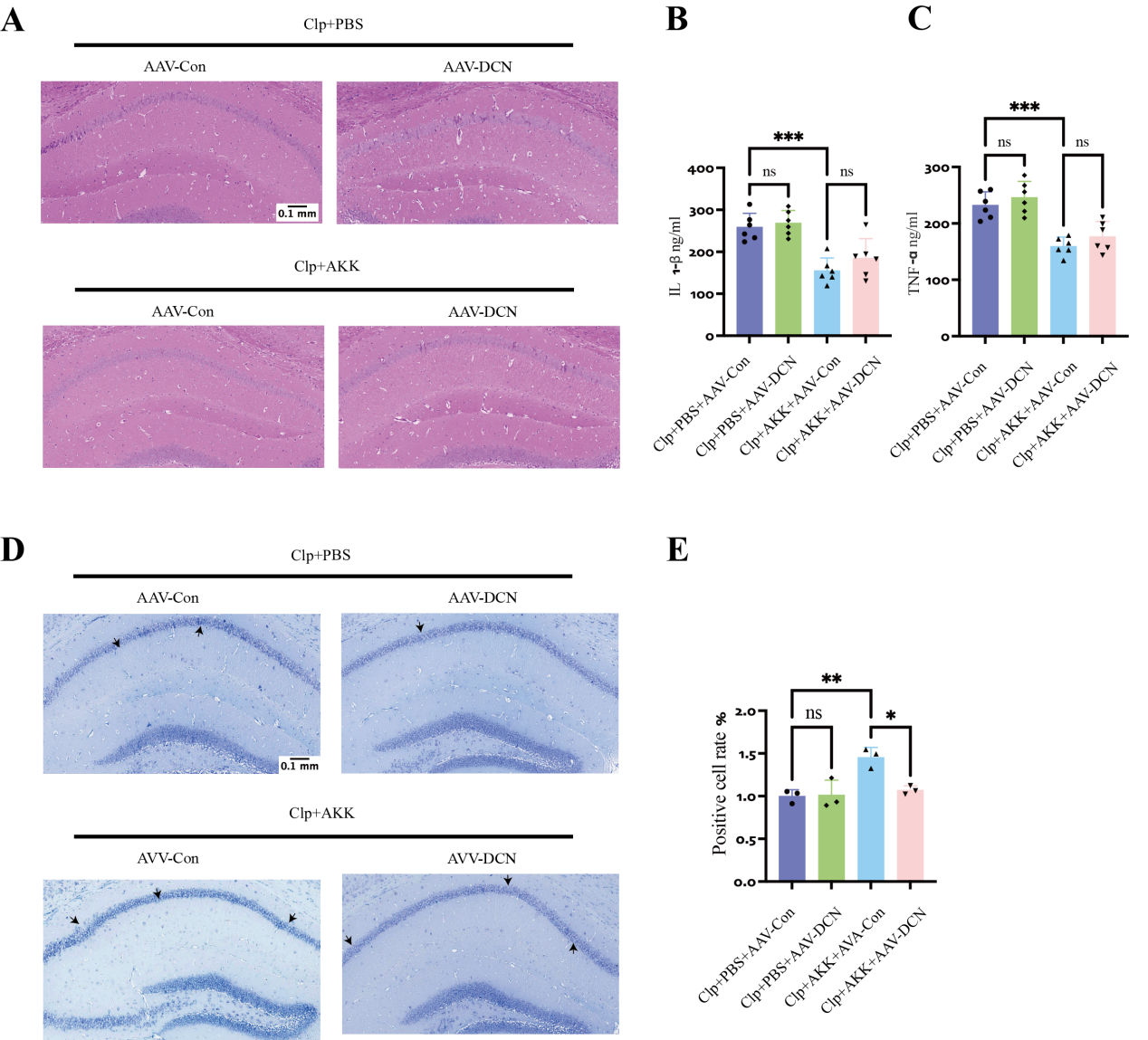


Supplementary Figure 3

Inhibition of DCN expression prevents AKK from exerting positive effects by reducing inflammation.

1. Representative H&HE-stained images (scale: 0.1 mm; magnification: × 20)(n=3). (B,C) Measurement of IL-1β and TNF-α levels in the hippocampi of septic mice (n=6). (D,E) Nissl-stained images of the hippocampal CA1 region of the mice and quantitative analysis of Nissl staining (scale: 0.1 mm, magnification: × 20) (n=3). The data are presented as the means ± SD. *p < 0.05, **p < 0.01, ***p < 0.001 and ****p < 0.0001.
